# A phase II study of preoperative chemoradiation with tegafur-uracil plus leucovorin for locally advanced rectal cancer with pharmacogenetic analysis

**DOI:** 10.1186/s13014-017-0800-5

**Published:** 2017-03-27

**Authors:** Sun Young Kim, Ji Yeon Baek, Jae Hwan Oh, Sung Chan Park, Dae Kyung Sohn, Min Ju Kim, Hee Jin Chang, Sun-Young Kong, Dae Yong Kim

**Affiliations:** 1 Center for Colorectal Cancer, Research Institute and Hospital, National Cancer Center, Ilsan-ro 323, Goyang-si, Gyeonggi-do 10408 Republic of Korea; 2 Department of Laboratory Medicine, Research Institute and Hospital, National Cancer Center, 323 Ilsan-ro, Ilsandong-gu, Goyang-si, Gyeonggi-do 10408 Republic of Korea; 3 Department of Oncology, Asan Medical Center, University of Ulsan College of Medicine, 88, Olympic-ro 43-gil, Songpa-gu, Seoul 05505 Republic of Korea

**Keywords:** Rectal neoplasms, Chemoradiotherapy, Tegafur, Uridine monophosphate synthetase

## Abstract

**Background:**

This study aimed to evaluate the efficacy of a high dose of oral tegafur-uracil (400 mg/m^2^) plus leucovorin with preoperative chemoradiation of locally advanced rectal cancer and to explore the impact of polymorphisms of cytochrome P 2A6 (*CYP2A6*), uridine monophosphate synthetase (*UMPS*), and ATP-binding cassette B1 (*ABCB1*) on clinical outcome.

**Methods:**

Patients with cT3 or cT4 rectal cancer were enrolled and were given tegafur-uracil 400 mg/m^2^/day and leucovorin 90 mg/m^2^/day for 7 days a week during preoperative chemoradiation (50.4 Gy/28 fractions) in this phase II trial. Primary endpoint was pathologic complete response rate, and the secondary endpoint was to explore the association between clinical outcomes and genetic polymorphisms *CYP2A6* (*4, *7, *9 and *10), *UMPS* G638C, and three *ABCB1* genotypes (C1236T, C3435T, and G2677T).

**Results:**

Ninety-one patients were given study treatment, and 90 underwent surgery. Pathologic complete response was noted in 10 patients (11.1%). There was no grade 4 or 5 toxicity; 20 (22.0%) experienced grade 3 toxicities, including diarrhea (10, 11.0%), abdominal pain (2, 2.2%), and anemia (2, 2.2%). Relapse-free survival and overall survival at 5 years were 88.6% and 94.2%, respectively. Patients with the *UMPS* 638 CC genotype experienced significantly more frequent grade 2 or 3 diarrhea (p for trend = 0.018).

**Conclusions:**

Preoperative chemoradiation with tegafur-uracil 400 mg/m^2^/day with leucovorin was feasible, but did not meet the expected pathologic complete response rate. The *UMPS* 638 CC genotype might be a candidate biomarker predicting toxicity in patients receiving tegafur-uracil/leucovorin-based preoperative chemoradiation for locally advanced rectal cancer.

**Trial registration:**

ISRCTN11812525, registered on 25 July 2016. Retrospectively registered.

## Introduction

Preoperative chemoradiation (CRT) with fluoropyrimidine such as 5-fluorouracil (5-FU) or capecitabine was shown to be effective in terms of reducing the risk of local recurrence of rectal cancer [1, 2], and has become the standard treatment. Tegafur-uracil (UFT) is another oral fluoropyrimidine that has shown similar efficacy to 5-FU as an adjuvant treatment for colorectal cancer [3]. It has also been tested as a preoperative CRT option for rectal cancer, but the doses and schedules have varied [4]. In general, UFT 300–400 mg/m^2^/day plus leucovorin (LV) 25–75 mg/day for 5 days a week at 45 Gy radiation (RT) for locally advanced rectal cancer was efficacious and tolerable [4]. This combination produced comparable outcomes to 5-FU in terms of toxicity profile and pathologic complete response rate in a randomized trial, although the study was underpowered due to incomplete accrual [5].

Many of the studies on UFT with CRT for rectal cancer were performed in a Caucasian population; however, the gastrointestinal toxicity of tegafur-based drugs such as UFT and S-1 is known to be more tolerable in Asian patients compared to Caucasians [6, 7]. This trend has not been fully explained by differences in pharmacokinetics or genetic polymorphisms.

On the premise of its favorable safety profile, increasing the dose of tegafur could be a strategy to enhance treatment efficacy in Asian patients. We obtained favorable results from a pilot preoperative CRT study with continuous dosing of high-dose (400 mg/m^2^/day) enteric-coated tegafur-uracil (UFT-E) and LV, which produced a pathologic complete response (pCR) rate of 22% in 36 patients [8]. Based on these results, we aimed to perform a phase II trial to evaluate the pCR rate and toxicity profile of preoperative CRT with UFT-E and LV.

To identify patients who benefit most from CRT with high-dose UFT-E with LV, individual difference in the process of metabolism and excretion of tegafur should be considered. *CYP2A6* and *UMPS* have crucial role in conversion of tegafur to active metabolite, and *ABCB1* encodes P-glycoprotein that pumps toxic metabolites out of gastrointestinal epithelium. With this phase II trial, we also planned to analyze trial participants’ genotypes for *CYP2A6*, *UMPS*, and *ABCB1*.

## Methods

### Patient eligibility

This study was designed as a single-center phase II trial evaluating pCR of UFT-E and LV with RT before total mesorectal excision (TME) of rectal cancer. Patients were eligible if they satisfied the following criteria: age ≥ 18 years; histologically confirmed adenocarcinoma of the rectum located within 8 cm of the anal verge by digital rectal exam; cT3-4 disease on magnetic resonance imaging (MRI)-based staging or rectal ultrasound; Eastern Cooperative Oncology Group (ECOG) performance status ≤ 2; adequate bone marrow, liver, and renal function. Patients were excluded if baseline imaging studies including computed tomography (CT) of chest, abdomen and pelvis led to suspicion of distant metastases, or if they had unresected synchronous colon cancer or a history of malignancy within 5 years before screening. The protocol of this study was approved by the Institutional Review Board of the National Cancer Center, Goyang, Korea (the protocol number NCCCTS-08-358). This study was conducted in accordance with the Declaration of Helsinki and Good Clinical Practice guidelines.

### Study treatment

CRT was started within 14 days after screening and obtaining informed consent. UFT-E was given orally as 400 mg/m^2^ of tegafur divided into three daily doses without drug holidays during RT. Since each package of UFT-E contains 500 mg of granules that corresponded to 100 mg of tegafur, the recommended dosing schedule according to body surface area (BSA) was as follows: BSA ≤ 1.37 m^2^: 2, 2, and 1 packages an hour after breakfast, lunch and dinner, respectively; BSA 1.38 m^2^ – 1.62 m^2^: 2, 2, and 2 packages; BSA 1.63 m^2^ – 1.87 m^2^: 3, 2, and 2 packages; and BSA ≥ 1.88 m^2^: 3, 3, and 2 packages. Each dose of UFT-E was administered with 2 15-mg tablets of LV, corresponding to a total daily dose of 90 mg.

Preoperative RT was delivered concurrently to the whole pelvis at a dose of 45 Gy in 25 fractions, followed by 5.4 Gy in a three-fraction boost to the primary tumor. The details of RT simulation, beam weights, and the RT field have been described previously [9]. Surgery was planned within 6 ± 2 weeks of the completion of CRT. TME was the first-choice surgical treatment, with the final decision regarding the choice of surgical procedure (abdominoperineal or anterior resection) made by the surgeon with the approval of a multidisciplinary team.

Postoperative chemotherapy was administered at discretion of the treating medical oncologist (SYK and JYB). One of the following regimens was administered for 4 months: 4 cycles of Mayo regimen 5-FU/LV (5-FU 400 mg/m^2^ and LV 20 mg/m^2^ on days 1–5, every 4 weeks), 3 or 4 cycles of UFT-E/LV (UFT-E 300 mg/m^2^ and LV 90 mg/day on day 1–28, every 5 weeks), or 8 cycles of FOLFOX-6 (oxaliplatin 85 mg/m^2^, LV 200 mg/m^2^, 5-FU bolus 400 mg/m^2^ and 5-FU continuous infusion 2400 mg/m^2^ for 46 h every 2 weeks).

### Evaluation

Clinical T and N staging according to the American Joint Committee on Cancer 7^th^ staging system by MRI or rectal ultrasound was recorded. The primary endpoint of this study, pCR, was evaluated according to Dworak’s classification [10] and the detailed procedure is described in our previous report [9]. A positive circumferential margin (CRM) was defined as tumor within ≤ 1 mm. The secondary endpoints included safety of CRT, relapse-free survival (RFS), overall survival (OS), and association of clinical outcomes with pharmacogenetic profile. RFS was calculated from the date of starting CRT to the date on which either of recurrence, progression, or death was first observed, or the date of last follow-up. OS was defined as from the date of starting CRT to the date of death from any cause or last follow-up. Safety outcomes were monitored according to the National Cancer Institute Common Terminology Criteria (NCI-CTC) scale, version 3.0.

### Protocol amendment

From January to June 2009, 23 patients were enrolled. Serious adverse events (admission due to toxicity) occurred in 6 patients (26.1%) and RT was interrupted due to grade 3 diarrhea in 2 patients. The unexpectedly high incidence of toxicity led to protocol amendment in July 2009: UFT-E and LV dosing days were reduced from 7 days to 5 days per week during CRT.

### Pharmacogenetic profiling

Genomic DNA was extracted from peripheral blood drawn before treatment. We assessed the presence of genetic variants of *CYP2A6*, *UMPS* and *ABCB1* using direct sequencing: CYP2A6*4 (whole deletion of *CYP2A6*), CYP2A6*7 (6558T > G, rs5031016), CYP2A6*9 (-48T > G, rs28399433), CYP2A6*10 (6558T > C and 6600G > T, rs28399468), *UMPS* 638G > C (rs1801019), *ABCB1* 3545 C > T (rs1045642), *ABCB1* 1236 C > T (rs1128503), and *ABCB1* 2677 G > T/A (rs2032582). Appropriate primers were designed and polymerase chain reaction (PCR) was performed using a GeneAmp PCR system 9700 thermal cycler (Applied Biosystems, Foster City, CA, USA). Sequencing was carried out with an Automated ABI Prism 3100 Genetic Analyzer (Applied Biosystems). The presence of the *CYP2A6* deletion allele (*4) was determined by restriction fragment length polymorphism (RFLP) as described in a previous study [11]. Positive (samples with known genotype) and negative control (water) were included to setting up sequencing reaction and each run of PCR-RFLP.

### Statistical analysis

We postulated that the pCR rate would be 20% or more, and the rate of no interest was less than 10%. Using Gehan-Simon’s two-stage phase II design implementing a type I error of 0.05 and a power of 90%, 55 patients would be accrued in stage 1, where study treatment was futile if less than 3 patients obtained pCR [12]. If pCR was observed in 4 or more patients in stage I, additional 54 patients would be enrolled. The primary endpoint would be considered to meet if pCR was achieved in 17 or more patients in stage II. Therefore, a total of 109 evaluable patients were needed, and the target sample size was 121 considering a drop-out rate of 10%.

The pCR rate was the proportion of patients who achieved pCR out of those who underwent TME with the 95% confidence interval (CI). RFS and OS at 3-years and 5-years follow-up were estimated with the Kaplan-Meier method and presented with the 95% CI. Safety and pCR rate were assessed according to different dosing schedules before and after protocol amendment and compared using Chi square test or Fisher’s exact test. The association of pharmacogenetic profile with clinical outcome was assessed with the Cuzick’s test for trend and log-rank test. As for *ABCB1*, analysis was done according to each single-nucleotide polymorphisms (SNPs) as well as the presence or absence of the reference allele haplotype*1 (1236C, 3435C, and 2677G). Logistic regression analysis was used for adjustment of clinical variables in order to ascertain the clinical impact of the pharmacogenetic profile. Statistical analysis was done using STATA 14.0 (StataCorp LP, College Station, TX)

## Results

### Patients

**Fig. 1:**
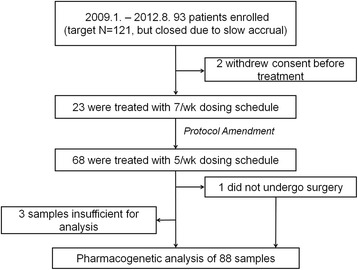
The flowchart of the clinical trial and pharmacogenetic analysis. Abbreviations:7/week, 7 days per week; 5/week, 5 days per week

From January 2009 to August 2012, 93 patients were enrolled. Enrollment was closed due to slow accrual in September 2012. Two patients withdrew consent before treatment; thus, a total of 91 patients were included, as shown in the study flowchart (Fig. 1). The baseline clinical characteristics of 91 patients are described in Table 1.

**Table 1:** Baseline Characteristics (*n* = 91)

Variables	N (%)
Age	Median 59 (range 33–75)
Sex	
Male	68 (74.33)
Female	23 (25.27)
ECOG PS	
0	83 (91.21)
1	8 (8.79)
Histologic differentiation	
Well differentiated	30 (32.97)
Moderately differentiated	60 (65.93)
Poorly differentiated	1 (1.10)
Clinical T stage	
cT3	88 (96.70)
cT4	3 (3.30)
Clinical N stage	
Negative	12 (13.19)
Positive	79 (86.81)
CEA	
< 5 ng/ml	58 (65.91)
≥ 5 ng/ml	30 (34.09)
Distance from anal verge	Median 6cm, range 1–8cm

*Abbreviation*: *ECOG PS* Eastern Cooperative Oncology Group performance status, *CEA* carcinoembryonic antigen

### Delivery of CRT

In the initial 23 (25.2%) patients who received UFT-E 400 mg/m^2^ 7 days per week (7/week cohort), the dose intensity was 92.6% of the intended dose, and the median cumulative and daily dose during RT was 14,074 mg/m^2^ and 370 mg/m^2^/day, respectively. After protocol amendment, 68 patients were treated with a 5-days per week dosing schedule (5/week cohort). The dose intensity was 98.9% of the intended dose and the median cumulative and daily dose was 11,073 mg/m^2^ and 291 mg/m^2^/day, respectively. Eighty-seven (95.6%) patients completed the planned RT schedule (50.4 Gy), while RT was interrupted in 4 patients (2 from the 7/week cohort and 2 from the 5/week cohort) due to grade 3 diarrhea. The mean of total RT dose was 50.1 Gy (range 41.4 – 50.4).

### Surgical procedures and pathologic response

TME was performed in 90 patients; one patient who was lost to follow-up after CRT eventually revisited our clinic with progression of the primary tumor and distant metastasis after 2 years. Eighty-three patients (92%) underwent surgery between 7.5 and 13.7 weeks (median 7 weeks) after completion of CRT. Two of 90 patients underwent synchronous resection of newly developed liver metastases that were noted during preoperative restaging procedures. A sphincter-saving procedure (low anterior resection or ultra-low anterior resection) was performed in 84 patients (93.3%). Another 6 patients underwent abdominoperineal resection. Laparoscopic surgery was done in 70 (77.8%). The CRM was positive (≤1mm) in 12 patients (13.3%), including 5 patients whose CRM was involved by tumor.

Ten patients achieved pCR (11.1%, 95% CI 5.4 – 19.5), 3 (13.0%) from the 7/week cohort and 5 (7.3%) from the 5/week cohort (odds ratio 1.28, 95% CI 0.30 – 5.45, *p* = 0.733). Downstaging to ypStage 0 or I was seen in 35 patients (38.9%), and the distribution of downstaging was similar between the two dosing cohorts; 9 (39.1%) from the 7/week cohort, 26 (38.2%) from the 5/week cohort; odds ratio 1.01, 95% CI 0.38 – 2.67, *p* = 0.978).

### Toxicity during and after CRT

There were no grade 4 or 5 adverse events among the 91 patients who received at least one dose of the study treatment, while 20 (22.0%) experienced grade 3 toxicity during CRT. Grade 2 or more diarrhea occurred in 14 patients (15.4%), 8 from the 7/week cohort (34.8%) and 6 (8.8%) from the 5/week cohort (odds ratio 5.51, 95% CI 1.66 – 18.29, *p* = 0.005). Stomatitis of grade 2 or greater also occurred more frequently in the 7/week cohort (17.4%) than in the 5/week cohort (3%; odds ratio 6.95, 95% CI 1.18 – 40.9, *p* = 0.032). The overall distribution of each adverse event is listed in Table 2.

**Table 2:** Adverse Events during Chemoradiation (*n* = 91)^a^

	Grade 1	Grade 2	Grade 3
Leukopenia	24 (26.37%)	11 (12.09%)	-
Neutropenia	1 (1.10%)	6 (6.59%)	-
Anemia	33 (36.26%)	7 (7.69%)	2 (2.20%)
Thrombocytopenia	6 (6.59%)	-	-
Fatigue	17 (18.68%)	-	-
Anorexia	43 (47.25%)	4 (4.40%)	1 (1.10%)
Nausea	41 (45.05%)	1 (1.10%)	-
Constipation	11 (12.09%)	1 (1.10%)	-
Diarrhea	11 (12.09%)	4 (4.40%)	10 (10.99%)
Stomatitis	10 (10.99%)	5 (5.49%)	1 (1.10%)
Abdominal pain	35 (38.46%)	7 (7.69%)	2 (2.20%)
Anal pain	13 (14.29%)	5 (5.49%)	-

^a^Adverse events were graded according to Common Terminology Criteria of Adverse Events version 3.0. There were no grade 4 or 5 adverse events

Acute postoperative complications within 30 days included anastomosis leakage (*n* = 9), urinary retention (*n* = 7), superficial incisional infection (*n* = 4), ileus (*n* = 4), bleeding (*n* = 2), deep vein thrombosis (*n* = 1), and pneumonia (*n* = 1). Among these complications, surgical intervention under spinal or general anesthesia was needed in 5 patients (3 for bleeding and 2 for anastomosis leakage). Delayed surgical intervention 30 days or more after TME was performed for anastomosis issues (leak, stricture or skin tag) in 8 patients who had undergone a sphincter-saving procedure; 5 underwent permanent stoma formation.

### Post-operative treatment and survival

Eighty-six patients were administered adjuvant chemotherapy for 4 months. Two patients with metastatic disease, 1 with poor health status (due to poor glycemic control and acute kidney injury) and 1 who was referred to another hospital and lost to follow-up did not receive adjuvant chemotherapy. Fluoropyrimidine monotherapy (5-FU/LV or UFT-E/LV) was given in 74 (86%) and the other 12 (14%) received FOLFOX-6.

**Fig. 2:**
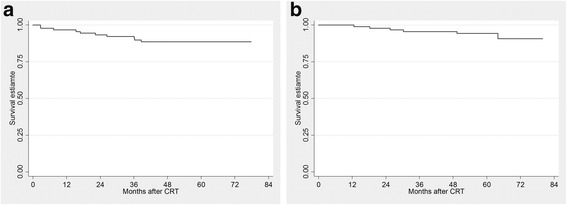
Relapse-free survival (**a**) and overall survival (**b**) of 91 patients who received study treatment

As of November 2015, 9 events (8 distant metastases and 1 local recurrence) had occurred; local curative treatment (metastasectomy or salvage CRT) was administered in 4 out of 9 recurred patients and all of them were alive without disease until the time of analysis. The patient who was lost to follow-up and later showed progression was censored at the time of follow-up loss in RFS analysis. With a median follow-up duration of 59.2 months (range 4.1 – 79.9), RFS at 3-years and 5-years follow-up was 92.2% (95% CI 84.3 – 96.2) and 88.6% (95% CI 79.9 – 93.7), respectively (Fig. 2A).

Six deaths occurred in patients who showed distant metastases. Five died of rectal cancer progression, and one patient with underlying emphysema died of pneumonia. OS at the 3-years and 5-years follow-up was 95.5% (95% CI 88.56 – 98.3%) and 94.2% (95% CI 86.57 – 97.55), respectively (Fig. 2B).

### Clinical outcome according to genotype

Pharmacogenenetic samples were obtained from 91 patients including a patient who did not undergo surgery, but 3 samples were insufficient for analysis (Fig. 1). Allelic frequencies of CYP2A6*4, *7, *9 and *10 were 0.12, 0.15, 0.21, and 0.08. Allelic frequencies for *UMPS* and *ABCB1* were 0.31, 0.4, 0.36, and 0.71 for *UMPS* G638C, *ABCB1* C1236T, *ABCB1* C3435T, and *ABCB1* G2677T, respectively. No significant deviations from Hardy-Weinberg equilibrium were seen except for *ABCB1* G2677T (*p* = 0.001). The occurrence of toxicity according to genotype is summarized in Table 3. As for *CYP2A6*, the presence of variant alleles (*4, *7, *9, or *10) was associated with leukopenia (p for tend = 0.022), but not with neutropenia (p for trend = 0.161). Grade 2 or greater stomatitis was only observed in variant homozygotes of *CYP2A6*. The presence of the *UMPS* G638C variant allele was associated with increased risk of diarrhea (p for trend = 0.018). SNPs or the presence of haplotype*1 of *ABCB1* were not associated with any type of toxicity.

**Table 3:** Adverse Events according to genotype (*n* =88)

Genotype	Leukopenia, ≥ grade 2	Neutropenia, ≥ grade 2	Anemia, ≥ grade 2	Diarrhea, ≥ grade 2	Anorexia, ≥ grade 2	Abd pain, ≥ grade 2	Stomatitis, ≥ grade 2	Any grade 3 toxicity
*CYP2A6*								
w/w (*n* = 22)	0 (0%)	0 (0%)	1 (4.6%)	3 (13.6%)	1 (4.6%)	4 (18.2%)	0 (0%)	7 (31.8%)
w/v (*n* = 37)	4 (10.8%)	3 (8.1%)	4 (10.8%)	4 (10.8%)	4 (10.8%)	2 (5.4%)	0 (0%)	6 (16.2%)
v/v (*n* = 29)	6 (20.7%)	3 (10.3%)	4 (13.8%)	7 (24.1%)	0 (0%)	3 (10.3%)	6 (15.8%)	7 (24.1%)
*P* value^a^	0.022^†^	0.161	0.291	0.269	0.398	0.428	0.012^†^	0.595
*UMPS* G638T								
GG (*n* = 41)	5 (12.2%)	3 (7.3%)	6 (14.6%)	4 (9.8%)	1 (2.4%)	2 (4.9%)	3 (7.3%)	8 (19.5%)
GC (*n* = 39)	5 (12.8%)	3 (7.7%)	2 (5.1%)	6 (15.4%)	3 (7.7%)	5 (12.8%)	2 (5.1%)	7 (18.0%)
CC (*n* = 8)	0 (0.0%)	0 (0.0%)	1 (12.5%)	4 (50.0%)	1 (12.5%)	2 (25.0%)	1 (12.5%)	5 (62.5%)
*P* value^a^	0.518	0.625	0.378	0.018^†^	0.183	0.067	0.557	0.078
*ABCB1* C1236T								
CC (*n* = 10)	1 (10.0%)	1 (10.0%)	1 (10.0%)	2 (20.0%)	0 (0.0%)	0 (0.0%)	0 (0%)	2 (20.0%)
CT (*n* = 49)	5 (10.2%)	2 (4.08%)	5 (10.2%)	7 (14.3%)	3 (6.1%)	5 (10.2%)	5 (10.2%)	12 (24.5%)
TT (*n* = 27)	4 (14.8%)	3 (11.1%)	3 (11.1%)	5 (18.5%)	2 (7.4%)	4 (14.8%)	1 (3.7%)	6 (22.2%)
*P* value^a^	0.584	0.584	0.902	0.914	0.459	0.214	0.900	0.985
*ABCB1* C3435T								
CC (*n* = 35)	3 (8.6%)	2 (5.7%)	2 (5.7%)	4 (11.4%)	2 (5.7%)	4 (11.4%)	2 (5.7%)	9 (25.7%)
CT (*n* = 42)	6 (14.3%)	3 (7.1%)	6 (14.3%)	9 (21.4%)	2 (4.8%)	4 (9.5%)	4 (9.5%)	9 (21.4%)
TT (*n* = 11)	1 (9.1%)	1 (9.1%)	1 (9.1%)	1 (9.1%)	1 (9.1%)	1 (9.1%)	0 (0%)	2 (18.2%)
*P* value^a^	0.717	0.689	0.447	0.723	0.804	0.776	0.819	0.559
*ABCB1* G2677T								
GG (*n* = 33)	3 (9.1%)	2 (6.1%)	2 (6.1%)	4 (12.1%)	2 (6.1%)	3 (9.1%)	2 (6.1%)	9 (27.3%)
GT (*n* = 26)	3 (11.5%)	2 (7.7%)	5 (19.2%)	5 (19.2%)	2 (7.7%)	3 (11.5%)	3 (11.5%)	5 (19.2%)
TT (*n* = 26)	3 (11.5%)	2 (7.7%)	2 (7.7%)	5 (19.2%)	1 (3.9%)	3 (11.5%)	1 (3.9%)	6 (23.1%)
*p* value^a^	0.754	0.802	0.754	0.450	0.745	0.754	0.797	0.678
*ABCB1* haplotype								
*1/*1 (*n* = 7)	1 (14.29%)	1 (14.29%)	0 (0%)	0 (0%)	0 (0%)	0 (0%)	0 (0%)	1 (14.3%)
*1/v (*n* = 36)	3 (8.33%)	1 (2.78%)	5 (13.89%)	3 (8.33%)	4 (11.11%)	4 (11.11%)	4 (11.11%)	10 (27.8%)
v/v (*n* = 42)	5 (11.90%)	4 (9.52%)	4 (9.52%)	2 (4.76%)	5 (11.90%)	5 (11.90%)	2 (4.76%)	9 (21.4%)
*P* value	0.872	0.727	0.872	0.573	0.966	0.756	0.477	0.925

*Abbreviations*: *CYP2A6* cytochrome P 2A6, *w* wild type allele, *v* variant allele, *Abd* abdominal, *UMPS* uridine monophosphate synthetase, *ABCB1* ATP-binding cassette B1

^a^test for trend by Cuzick’s test

^†^ *P* value <0.05

Since the different dosing schedules used could have affected the incidence of toxicity in this study, especially diarrhea, the impact of polymorphisms on any toxicity ≥ grade 3 was tested again with adjustment for the dosing schedule as well as age, sex and performance status (Table 4); the CC genotype was the only genotype that was associated with increased risk of grade 3 or more toxicity. It was also significantly associated with grade 2 or greater diarrhea (odds ratio = 10.8, 95% CI 1.50 – 77.40, *p* = 0.018) after adjustment, while the GC genotype did not have a significant association with toxicity compared to the GG genotype (odds ratio = 1.96, 95% CI 0.42 – 9.06, *p* = 0.389). Goodness-of-fit test suggested the fit of recessive model for association of *UMPS* with diarrhea (*p* = 0.111) was better than that of additive model (*p* = 0.056).

**Table 4:** Multivariable analysis of risk for toxicity for patients carrying variant alleles

Genotype		Diarrhea, ≥ Grade 2			Any grade 3 toxicity		
		Adjusted OR^a^	95% CI	P value	Adjusted OR^a^	95% CI	P value
*CYP2A6*	w/w (*n* = 22)	1 (reference)			1 (reference)		
	w/v (*n* = 37)	0.69	0.12 – 3.89	0.671	0.29	0.07 – 1.19	0.086
	v/v (*n* = 29)	1.87	0.38 – 9.29	0.443	0.52	0.13 – 2.03	0.343
*UMPS* G638T	GG (*n* = 41)	1 (reference)			1 (reference)		
	GC (*n* = 39)	1.96	0.42 – 9.06	0.389	0.97	0.28 – 3.40	0.962
	CC (*n* = 8)	10.76	1.50 – 77.39	0.018^†^	10.2	1.44 – 72.13	0.020^†^
*ABCB1* C1236T	CC (*n* = 10)	1 (reference)			1 (reference)		
	CT (*n* = 49)	0.43	0.06 – 3.13	0.406	0.99	0.16 – 5.97	0.990
	TT (*n* = 27)	1.18	0.15 – 9.37	0.873	1.14	0.17 – 7.83	0.891
*ABCB1* C3435T	CC (*n* = 35)	1 (reference)			1 (reference)		
	CT (*n* = 42)	2.03	0.50 – 8.33	0.324	0.61	0.19 – 1.99	0.410
	TT (*n* = 11)	1.10	0.10 – 12.19	0.940	0.82	0.13 – 5.11	0.836
*ABCB1* G2677T	GG (*n* = 33)	1 (reference)			1 (reference)		
	GT (*n* = 26)	1.45	0.30 – 6.92	0.645	0.44	0.11 – 1.77	0.248
	TT (*n* = 26)	1.89	0.40 – 8.86	0.420	0.80	0.22 – 2.92	0.737
*ABCB1* haplotype	*1/*1 (*n* = 7)	1 (reference)			1 (reference)		
	*1/v (*n* = 36)	1.14	0.11 – 12.15	0.915	1.68	0.17 – 16.9	0.662
	v/v (*n* = 42)	0.51	0.04 – 6.06	0.591	1.46	0.15 – 14.6	0.747

*Abbreviations*: *OR* odds ratio, *CI* confidence interval, *CYP2A6* cytochrome P 2A6, *w* wild type allele, *v* variant allele, *UMPS* uridine monophosphate synthetase, *ABCB1* ATP-binding cassette B1

^a^Adjusted for dosing schedule (7 days/week vs. 5 days/week), age, sex, and ECOG performance status (0 vs 1)

^†^ *P* value <0.05

Genotype and pCR rate analysis revealed that nonsignificant relationship: there was a trend of increasing pCR rate according to the number of *UMPS* G638C allele (4.9% in GG, 15.8% in GC and 25.0% in CC) but not statistically significant (*p* = 0.05). Neither was seen any ordinal relationship of pCR rate with other genotypes: *CYP2A6* (*p* = 0.174), *ABCB1* C1236T (*p* = 0.094), C3435T (*p* = 0.949), *ABCB1* G2677T (*p* = 0.406), and *ABCB1* variant haplotype other than *1 (*p* = 0.302). RFS was not also associated with any genotypes (data not shown).

## Discussion

Our study indicated that preoperative CRT with UFT-E was feasible in Korean patients with locally advanced rectal cancer and that *UMPS* polymorphisms might be predictive of UFT - based CRT toxicity.

The observed pCR rate of 11% is consistent with other CRT studies based on 5-FU or capecitabine [2], although it did not meet the primary endpoint, which was expected to be more than 20%. The endpoint was set based on our pilot study, where the pCR rate was as high as 22% (95% CI 10.1–39.2), with 8 of 36 patients achieving pCR. We can exclude the possibility of the pCR rate being affected by the number of sections or inter-observer variation of the examining pathologists; surgical specimens were examined according to consistent sectional criteria by a single pathologist (HJC) in both the pilot study and this phase II trial.

We postulated that an increased dose of UFT-E might improve the pCR rate, but the results of this study were insufficient to suggest such a dose-response relationship. Furthermore, the pCR rate was similar between the 7/week and 5/week cohorts. Intensifying chemotherapy with oxaliplatin also did not seem to be a successful method of improving pCR or disease-free survival in phase III trials [13–16]. A dose of 400 mg/m^2^/day of UFT-E was chosen based on phase I trial data indicating that continuous dosing with 400 mg/m^2^/day administered as 3 divided doses was tolerable [17], as well as our pilot study showing a tolerable safety profile (grade 3 or 4 diarrhea in 12.8% of patients) with 400 mg/m^2^/day 7 days per week. Eventually the dosing schedule was modified due to toxicity; it was similar strategy in NSABP R-04 trial, where the dosing days of capecitabine or 5-FU were reduced from 7 to 5 days per week due to toxicity concerns [18]. Grade 3 diarrhea developed in 26.1% of the 7/week cohort, whereas only 5.9% of the 5/week cohort experienced it in our trial. The R-04 trial also showed that the incidence of grade 3 to 5 diarrhea decreased from 16 to 7% after protocol amendment [18].

Nevertheless, our study showed that UFT-E 400 mg/m^2^/day for 5 days per week with LV was feasible for preoperative CRT for locally advanced rectal cancer. The highest dose of UFT with RT in previous studies was 400 mg/m^2^, but it was not accompanied by LV, which enhances the activity of 5-FU by promoting formation of covalent bonds with thymidylate synthase, the target enzyme of 5-FU [19].

Our study showed that *UMPS* polymorphisms might be related to a higher incidence of diarrhea; an ordinal relationship between grade 2 or 3 diarrhea and the number of G638C variant alleles was shown. These results could be explained by metabolism of 5-FU; metabolites of 5-FU by *UMPS* cause gastrointestinal toxicity via incorporation into RNA (F-RNA) (20). *UMPS* G638C is a polymorphism that reportedly confers enhanced enzymatic activity; grade 3–4 diarrhea in 5-FU or UFT/LV occurred more frequently in Japanese patients with *UMPS* G538C genotype (20, 21). It should be noted pharmacogenetic studies on association of *UMPS* genotype and fluoropyrimidines including ours were only exploratory analyses, not confirmative biomarker study; there are still insufficient evidences on genotype-guided fluoropyrimidine dosing in practice [20]. Furthermore, in this study, we cannot rule out the possibility that these results were confounded by other clinical variables, especially the varied dosing schedule (7/week versus 5/week), although the CC genotype was still a significant predictor of toxicity after adjustment for the clinical variables including dosing schedule.

Clinical activity of 5-FU or UFT was associated with enhanced enzymatic activity or higher expression of *UMPS* in several studies [21, 22], thus CC genotype harbor a possibility of resulting improved clinical outcome. In our study, pCR rate tended to increase according to the number of G638C variant allele without statistical significance, while RFS was not related to the *UMPS* polymorphism. Although this study did not have adequate power to show the relationship of anti-tumor activity of UFT-E and *UMPS* polymorphism, improved pCR rate in *UMPS* 638 CC genotype (25%) could be considered for further investigation.

Toxicity of tegafur-based drugs such as S-1 and UFT occurs more frequently in Caucasian than in Asian patients, which was the rationale for the higher dose of UFT-E used in our study. Differences in pharmacokinetic exposure to active metabolite (5-FU) did not explain this phenomenon, suggesting that a more complicated mechanism might underly [6, 7]. Reduced biotransformation of tegafur to 5-FU by the variant *CYP2A6*, which is harbored more frequently by Asian patients, was thought to be one of the causes of ethnic differences, but such a relationship was not clear in other studies with S-1-based regimen [23–25]. In this study, leukopenia and stomatitis were more frequent in variant genotypes, which were unexpected findings. However, this is consistent with the case report of a patient with the CYP2A6*9 allele who showed severe UFT toxicity; the authors suggested tegafur may be alternatively metabolized by cytosolic enzymes as a mechanism of increased toxicity [26]. The impact of *CYP2A6* genotype on tegafur-based drugs needs to be clarified through well-designed prospective trials.

The gene product of *ABCB1*, P-glycoprotein, removes toxic metabolites from cells; hence, it can also induce chemo-resistance in malignant cells. It is extensively expressed in the gastrointestinal epithelium and might control the uptake of oral agents such as UFT from the gut [27]. Although fluoropyrimidines are not a known substrate of ABCB1, ABCB1 expression is induced along with 5-FU resistance in some cell lines [28], which suggests polymorphisms concerning ABCB1 activity might be related to efficacy of fluoropyrimidines. Otherwise, *ABCB1* haplotype*1 was reportedly associated with capecitabine toxicity compared to variant haplotypes [29]. However, a significant relationship between *ABCB1* polymorphisms and clinical outcome was not observed in this study.

This study has several limitations; we stopped enrollment due to slow accrual, resulting in insufficient statistical power for the pCR rate of CRT with UFT-E. The association between toxicity profile and genotype was not supported by the pharmacokinetic study. We explored polymorphisms of 3 genes (*UPMS*, *CYP2A6*, and *ABCB1*) which relate to metabolism and excretion of tegafur or 5-FU, but other genes such as *TYMS* and *DPYD,* which are well-known predictors of 5-FU toxicity [30, 31], should have been tested. Our pilot study with a higher dose of UFT-E should have been done with a meticulous dose-escalating scheme. Different dosing schedule before and after protocol amendment was a major confounding factor in the interpretation of efficacy and safety outcomes, as well as of the pharmacogenetic study.

## Conclusions

UFT-E 400 mg/m^2^/day with LV for 5 days per week was shown to be a feasible regimen when administered with preoperative RT for patients with locally advanced rectal cancer. A SNP of *UMPS*, G638C, was predictive of UFT-E toxicity and could be studied further to explore genotype-guided dosing.

## Abbreviations

5-FU: 5-fluorouracil
ABCB1: ATP-binding cassette B1
CI: Confidence interval
CRM: Circumferential margin
CRT: Chemoradiation
CT: Computed tomography
CYP2A6: Cytochrome P 2A6
ECOG: Eastern Cooperative Oncology Group
LV: Leucovorin
MRI: Magnetic resonance imaging
NCI-CTC: National Cancer Institute Common Terminology Criteria
OS: Overall survival
pCR: Pathologic complete response
RFS: Relapse-free survival
RT: Radiation
SNP: Single-nucleotide polymorphisms
TME: Total mesorectal excision
UFT: Tegafur-uracil
UFT-E: Enteric-coated tegafur-uracil
UMPS: Uridine monophosphate synthetase
